# Association of physical activity and sedentary time with structural brain networks—The Maastricht Study

**DOI:** 10.1007/s11357-020-00276-z

**Published:** 2020-10-09

**Authors:** Laura W. M. Vergoossen, J. F. A. Jansen, J. J. A. de Jong, C. D. A. Stehouwer, N. C. Schaper, H. H. C. M. Savelberg, A. Koster, W. H. Backes, M. T. Schram

**Affiliations:** 1 Department of Radiology & Nuclear Medicine, Maastricht University Medical Center+, Maastricht, The Netherlands; 2 School for Mental Health and Neuroscience (MHeNs), Maastricht University, Maastricht, The Netherlands; 3 School for Cardiovascular Disease (CARIM), Maastricht University, Maastricht, The Netherlands; 4 Department of Electrical Engineering, Eindhoven University of Technology, Eindhoven, The Netherlands; 5 Care and Public Health Research Institute (CAPHRI), Maastricht University, Maastricht, The Netherlands; 6 Department of Nutrition and Movement Sciences, NUTRIM School of Nutrition and Translational Research in Metabolism, Maastricht University, Maastricht, The Netherlands; 7 Department of Social Medicine, Maastricht University, Maastricht, The Netherlands; 8 Heart and Vascular Centre, Maastricht University Medical Center+, Maastricht, the Netherlands; 9 Department of Internal Medicine, Maastricht University Medical Center+, PO Box 5800, AZ 6202 Maastricht, The Netherlands

**Keywords:** Physical activity, Sedentary behavior, Structural connectivity, Elderly population–based cohort

## Abstract

We assessed whether objectively measured low- and high-intensity physical activity (LPA and HPA) and sedentary time (ST) were associated with white matter connectivity, both throughout the whole brain and in brain regions involved in motor function. In the large population-based Maastricht Study (*n* = 1715, age 59.6 ± 8.1 (mean ± standard deviation) years, and 48% women), the amounts of LPA, HPA, and ST were objectively measured during 7 days by an activPAL accelerometer. In addition, using 3T structural and diffusion MRI, we calculated whole brain node degree and node degree of the basal ganglia and primary motor cortex. Multivariable linear regression analysis was performed, and we report standardized regression coefficients (stβ) adjusted for age, sex, education level, wake time, diabetes status, BMI, office systolic blood pressure, antihypertensive medication, total-cholesterol-to-HDL-cholesterol ratio, lipid-modifying medication, alcohol use, smoking status, and history of cardiovascular disease. Lower HPA was associated with lower whole brain node degree after full adjustment (stβ [95%CI] = − 0.062 [− 0.101, − 0.013]; *p* = 0.014), whereas lower LPA (stβ [95%CI] = − 0.013 [− 0.061, 0.034]; *p* = 0.580) and higher ST (stβ [95%CI] = − 0.030 [− 0.081, 0.021]; *p* = 0.250) was not. In addition, lower HPA was associated with lower node degree of the basal ganglia after full adjustment (stβ [95%CI] = − 0.070 [− 0.121, − 0.018]; *p* = 0.009). Objectively measured lower HPA, but not lower LPA and higher ST, was associated with lower whole brain node degree and node degree in specific brain regions highly specialized in motor function. Further research is needed to establish whether more HPA may preserve structural brain connectivity.

**Electronic supplementary material:**

The online version of this article (10.1007/s11357-020-00276-z) contains supplementary material, which is available to authorized users.

## Introduction

It is increasingly acknowledged that low physical activity is harmful not only for general health [19] but also for the brain [9, 13, 16]. In addition, sedentary behavior, which is a risk factor independent of physical activity, may also be associated with cognitive decline [7, 36]. However, how both physical activity and sedentary behavior exactly affect early changes in brain function is not yet clear.

A growing body of evidence shows a clear association between low physical activity and sedentary behavior and structural brain changes, such as brain atrophy [1, 5, 11, 22] and cerebral small vessel disease (cSVD) [38] at the population level. However, both atrophy and cSVD are likely to represent irreversible damage, while novel markers of early reversible brain changes may be available. Previous studies have indicated that the structural organization of brain networks, also named connectivity, may represent such a marker, which is also affected in dementia [25]. However, data on the association between physical activity and structural brain networks are scarce [14], while the association of sedentary behavior and structural brain networks have not been reported yet.

In this study, we hypothesize that both whole brain structural organization of brain networks and the organization of specific regions involved in motor function, as the basal ganglia and the primary motor cortex [2], are affected by low physical activity and high levels of sedentary time. Therefore, we assessed the association of objectively measured low- and high-intensity physical activity and sedentary time with both whole brain and regional white matter structural connectivity within the population-based Maastricht Study.

## Research design and methods

### The Maastricht Study: population and design

We used data from The Maastricht Study, an observational prospective population-based cohort study. The rationale and methodology have been described previously [20]. In brief, the study focuses on the etiology, pathophysiology, complications, and comorbidities of type 2 diabetes mellitus (T2DM) and is characterized by an extensive phenotyping approach. Eligible for participation were all individuals aged between 40 and 75 years and living in the southern part of the Netherlands. Participants were recruited through mass media campaigns and from the municipal registries and the regional Diabetes Patient Registry via mailings. Recruitment was stratified according to known T2DM status, with an oversampling of individuals with T2DM, for reasons of efficiency. The present report considered cross-sectional data from the first 3451 participants, who completed the baseline survey between November 2010 and September 2013. The examinations of each participant were performed within a time window of three months (Supplementary Figure 1). MRI measurements were implemented from December 2013 onwards until February 2017 and were available in 2318 out of 3451 participants. Of the 2318 participants with MRI measurements available, 2302 participants had complete data without artifacts, and 1715 participants of those also had objectively measured physical activity data available (Flowchart in Supplementary material Figure 2). The study has been approved by the institutional medical ethical committee (NL31329.068.10) and the Minister of Health, Welfare and Sports of the Netherlands (Permit 131088-105234-PG). All participants gave written informed consent.

### Physical activity and sedentary time measures

Daily activity levels were measured using the activPAL3™ physical activity monitor (PAL Technologies, Glasgow, UK). This device is a small (53 × 35 × 7 mm), lightweight (15 g) triaxial accelerometer that records movement in the vertical, anterio-posterior, and mediolateral axes and also determines posture (sitting or lying, standing, and stepping) based on acceleration information. The device was attached directly to the skin on the front of the right thigh with (transparent 3M Tegaderm™) tape, after the device had been waterproofed using a nitrile sleeve. Participants were asked to wear the accelerometer for 8 consecutive days, without removing it at any time. To avoid inaccurately identifying non-wear time, participants were asked not to replace the device once removed. The method for determining the waking time has been described elsewhere [31]. Data were uploaded using the activPAL software and processed using customized software (MATLAB R2013b, MathWorks, Natick, MA, USA). Data from the first day were excluded from the analysis because participants performed physical function tests at the research center after the device was attached. In addition, data from the final wear day providing ≤ 14 waking hours of data were excluded from the analysis. Participants were included if they provided at least one valid day (≥ 10 h of waking data).

The total amount of stepping time was based on the stepping posture and calculated as the mean time spent in that position during waking time per day [30]. The total stepping time was further classified into high-intensity physical activity (HPA, minutes with a step frequency > 110 steps/min during waking time) [26] and low-intensity physical activity (LPA, minutes with a step frequency ≤ 110 steps/min during waking time). In this study, we aimed to identify risk factors for brain alterations. As physical activity may be protective and sedentary time a risk, we choose to consider high physical inactivity (low physical activity) and high sedentary time as risk factors. Therefore, we inversed the physical activity data (e.g., multiplied by – 1) and the inverse of total, low-intensity, and high-intensity physical activity was used in statistical analyses to represent low physical activity levels. The total amount of sedentary time (ST) was based on the sedentary posture (sitting or lying) and calculated as the mean time spent in a sedentary position during waking time per day.

For descriptive purposes, we present the data in Table 1 according to the recently published physical activity guidelines [17, 35]. These guidelines both recommend at least 150 min of high-intensity physical activity per week for considerable health benefits, including brain health. Participants that were compliant with these guidelines were indicated as the high HPA group, and those that were not compliant as the low HPA group. We used these categories to assess the reference networks for white matter integrity to address potential differences in connectivity between active and inactive participants.

**Table 1:** General characteristics of participants stratified by low or high HPA measured by the ActivPAL

Characteristic	Total (*n* = 1715)	High HPA (*n* = 780)	Low HPA (*n* = 935)	*P*
Demographics				
Age (years)	59.6 ± 8.1	58.0 ± 7.9	60.9 ± 8.0	< 0.001
Sex, female (No. [%])	830 [48.4]	447 [57.3]	383 [41.0]	< 0.001
Education level (No. [%]), low/middle/high	523/497/695 [30.5/29.0/40.5]	225/224/331 [28.9/28.7/42.4]	297/274/364 [31.8/29.3/38.9]	0.283
Cardiovascular risk factors				
BMI (kg/m^2^)	26.6 ± 4.2	25.5 ± 3.7	27.6 ± 4.4	< 0.001
Waist circumference (cm)	94.4 ± 12.7	89.8 ± 11.0	98.2 ± 12.8	< 0.001
Systolic blood pressure (mmHg)	134.0 ± 17.3	131.8 ± 16.9	135.8 ± 17.4	< 0.001
Diastolic blood pressure (mmHg)	76.1 ± 9.7	75.3 ± 9.8	76.7 ± 9.6	0.003
T2DM (No. [% of T2DM])	400 [23.6]	99 [12.9]	301 [32.6]	< 0.001
Hypertension, yes (No. [%])	914 [53.3]	345 [44.2]	569 [60.8]	< 0.001
Total-to-HDL-cholesterol ratio	3.6 ± 1.1	3.3 ± 1.0	3.7 ± 1.2	< 0.001
History of CVD, yes (No. [%])	218 [12.7]	57 [7.3]	161 [17.2]	< 0.001
Medication use				
Diabetes medication, yes (No. [%])	308 [18.0]	72 [9.2]	236 [25.2]	< 0.001
Antihypertensive medication, yes (No. [%])	619 [36.1]	199 [25.6]	420 [44.9]	< 0.001
Lipid-modifying medication, yes (No. [%])	540 [31.5]	178 [22.9]	362 [38.7]	< 0.001
Lifestyle factors				
Alcohol (No. [%]), none/low/high	291/980/444 [17.0/57.2/25.9]	108/452/220 [13.8/58.0/28.2]	183/528/224 [19.6/56.5/23.9]	0.003
Smoking (No. [%]), never/former/current	651/854/210 [38.0/49.8/12.3]	326/380/74 [41.7/48.8/9.6]	325/474/136 [34.8/50.7/14.5]	0.001
Diet (Dutch Healthy Diet, 0-100)^‡^	85.6 ± 14.3	83.1 ± 14.8	82.3 ± 14.0	< 0.001
Total PA (min/day)	124 ± 41	145 ± 37	106 ± 35	< 0.001
LPA (min/day)	100 ± 33	106 ± 31	95 ± 34	< 0.001
HPA (min/day)	24 ± 19	39 ± 18	11 ± 6	< 0.001
Sedentary time (min/day)	558 ± 99	530 ± 93	581 ± 99	< 0.001
Wake time (min/day)	945 ± 53	951 ± 50	940 ± 54	< 0.001
Cognitive score				
MMSE total score	29.0 ± 1.2	29.2 ± 1.1	28.9 ± 1.2	< 0.001
Node degree				
Whole brain	20.9 ± 0.8	21.1 ± 0.7	20.8 ± 0.8	< 0.001
Frontal lobe	17.8 ± 0.9	17.9 ± 0.9	17.7 ± 1.0	< 0.001
Temporal lobe	19.8 ± 0.9	19.9 ± 0.8	19.7 ± 0.9	< 0.001
Parietal lobe	20.3 ± 0.8	20.3 ± 0.8	20.4 ± 0.9	0.150
Occipital lobe	24.4 ± 1.3	24.4 ± 1.3	24.3 ± 1.3	0.354
Basal ganglia	42.0 ± 2.0	42.3 ± 2.0	41.8 ± 2.0	< 0.001
Primary motor cortex	25.0 ± 2.7	25.3 ± 2.6	24.8 ± 2.8	0.001
Other				
MRI lag time (years)	2.0 ± 1.2	2.0 ± 1.2	2.1 ± 1.2	0.097

General characteristics of the study population were evaluated by ANOVA (continuous variables with a normal distribution) or χ2 tests (categorical variables). Data are presented as means ± standard deviation or percentages for categorical variables. High HPA was defined as at least 150 min of high-intensity physical activity per week (21.4 min per day)

*No.* number, *PA* physical activity, *T2DM* type 2 diabetes mellitus, *HDL* high-density lipoprotein, *CVD* cardiovascular disease, *MMSE* Mini-Mental State Examination

^‡^Diet score was available in *n* = 1613

### Magnetic resonance imaging

Magnetic resonance imaging (MRI) was performed on a 3T MRI scanner (MAGNETOM Prisma-fit Syngo MR D13D, Siemens Healthcare, Erlangen, Germany) by use of a 64-element head/neck coil for parallel imaging with an acceleration factor of two. A 3D T1-weighted magnetization prepared rapid acquisition gradient echo (MPRAGE) sequence (TR/TI/TE 2300/900/2.98 ms, 176 slices, 256 × 240 matrix size, and 1.00 mm cubic voxel size) was acquired for anatomic reference. Diffusion-weighted MRI (dMRI) data were acquired with a diffusion sensitized echo-planar imaging (EPI) sequence (TR/TE 6100/57 ms, 65 slices, 100 × 100 matrix size, 2.00 mm cubic voxel size, and 64 diffusion sensitizing gradient directions (*b* = 1200 s/mm^2^)). In addition, three minimally diffusion-weighted images (*b* = 0 s/mm^2^) were acquired.

### Image preprocessing

To define *N* = 120 brain regions, the Automatic Anatomical Labeling (AAL2) atlas [27] was used. The atlas volumes of interest were transformed to diffusion image space for each individual subject. First, affine registrations of the dMRI image to the T1 image and of the T1 image to T1 Montreal Neurological Institute-152 standard space [8] were performed. These two transformations were combined, and the inverse transformation matrix was applied to the AAL2 template. T1-weighted images were segmented by use of a certified (ISO13485:2012), automated method (which included visual inspection) [3, 34]. T_1_-weighted images were segmented into gray matter, white matter, and cerebrospinal fluid volumes (1 voxel = 1.00 mm^3^ = 0.001 ml) [34]. Intracranial volume was calculated as the sum of gray matter, white matter, and cerebrospinal fluid volumes. Total brain parenchyma volume was calculated as the sum of gray and white matter volumes. dMRI (pre)processing was performed with the diffusion MR Toolbox ExploreDTI version 4.8.6 [15]. The main preprocessing steps were eddy current induced geometric distortions and head motion correction and estimation of the diffusion tensor. After preprocessing, fiber orientation distributions (FOD) were estimated using constrained spherical deconvolution with a maximum harmonic degree of 8, which allows fiber tracking through regions with crossing fibers [23]. Whole brain deterministic tractography was performed using FOD sampling [12] with a seed point resolution of 2 mm^3^, a step size of 1 mm, and FOD and maximum deflection angle threshold of 0.1 and 30°, respectively. The next step was to perform connectivity analysis to obtain white matter tracts from and to all the AAL2 brain regions. A previous study of our group confirmed the robustness of tract volume as a measure for the edge weighting [28]; therefore, for each connection, the tract volume was calculated as the number of voxels visited by at least one tract between the areas concerned multiplied by the voxel volume (in mm^3^) (as previously described [32]). The obtained connectivity matrix with tract volumes was normalized to intracranial volume to reduce inter-subject variation [37]. When regions were connected by only one or two streamlines, the corresponding tract volumes were removed from the connectivity matrix, as an additional noise filter.

### White matter networks

Network analysis was performed using the Brain Connectivity Toolbox (version 2017-15-01) [18] in MATLAB (Release 2016a, The MathWorks, Inc., Natick, Massachusetts, USA). In this method, the brain was represented as a graph, which is a network of nodes (i.e., gray matter brain regions) connected by edges (i.e., white matter connections between brain regions). The node degree was calculated for each atlas region, and the mean value was defined as the whole brain node degree, which is a measure for the average number of edges connected to a node. In a network with a high whole brain node degree, brain regions are connected to many other brain regions in the network (i.e., strong innervation). The sparsity of a network is the ratio of the number of missing connections in a network to the possible number of connections and is closely, but inversely, related to the node degree. The sparsity ranges from 0 to 1, the higher the sparsity, the lower the density of the network [18].

Subsequently, reference networks were calculated [4] based on predefined levels of physical activity and sedentary time. Note that these reference networks may differ per group comparison. The reference networks were proportionally thresholded to a sparsity of 0.80 (only the 20% connections with the highest occurrence in the individual connectivity matrices of the participants in that group), resulting in a weighted, undirected network with a sparsity close to the sparsity of the standard network (more detailed information can be found in the Supplementary Material).

**Fig. 1:**
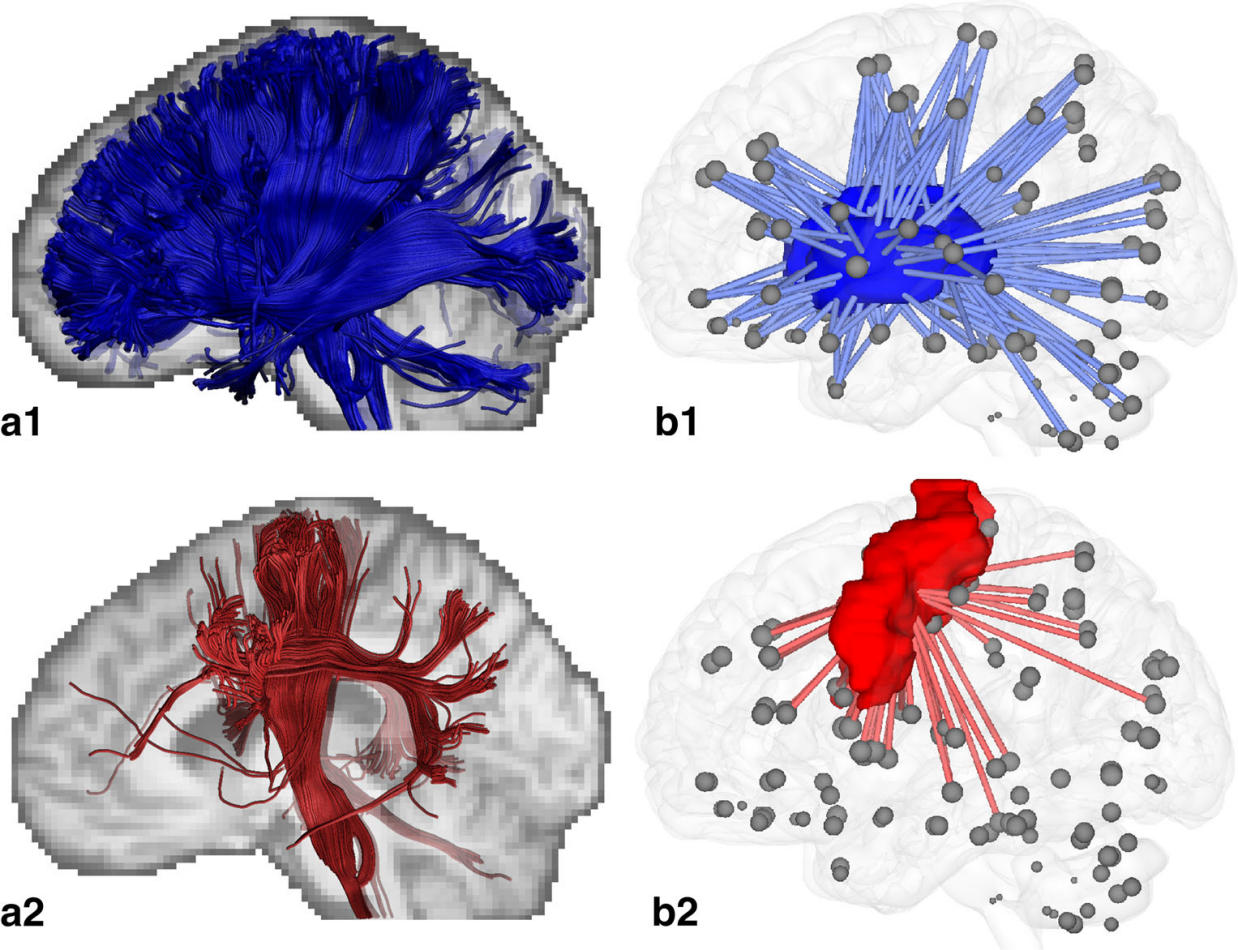
White matter tracts crossing the basal ganglia and primary motor cortex. Tracts crossing the basal ganglia (A1) are mainly projection fibers, and crossing the primary motor cortex (A2) pyramidal tracts (e.g., the cortico-spinal tract). Blue and red volumes indicate the locations of the basal ganglia (B1) and primary motor cortex (B2), respectively. The gray dots represent the centers of 120 atlas regions. Lines indicate connections from a subset of brain regions to these two volumes; the number of lines is equal to the node degree

The first reference network was based on the high HPA group (participants who met the physical activity guidelines [17, 35], i.e., at least 150 min of high-intensity physical activity per week (21.4 min per day)). With regard to sedentary behavior, as mentioned above, the guidelines only advise reducing sitting without providing numbers. Therefore, the sedentary behavior reference network was based on the tertile of the participants with the lowest sedentary time (i.e., less than 512 min of sedentary time per day). Thereafter, we calculated the whole brain node degree for both reference networks. To investigate local connectivity changes, we calculated the node degree for brain regions important for physical activity and motor functions, i.e., the basal ganglia (i.e., caudate nucleus, putamen, pallidum, and thalamus as defined by the AAL2 atlas) (Fig. 1A) and the primary motor cortex (Fig. 1B), and for the four brain lobes (i.e., the frontal (without primary motor cortex), temporal, parietal, and occipital lobe).

### General characteristics and covariates

Educational level (low, intermediate, high), smoking status (never, current, former), and history of cardiovascular disease were assessed by questionnaires [21]. Medication use was assessed in an interview where generic name, dose, and frequency were registered. We measured weight, height, BMI, waist circumference, blood pressure (measured in office [Omron 705IT, Japan]), and plasma lipid profile [21].

### Statistical analysis

All statistical analyses were performed by use of the Statistical Package for Social Sciences (SPSS Statistics 23.0, IBM, Chicago, IL, USA). Multivariable linear regression analysis was used to investigate the association of physical activity and sedentary behavior, with whole brain node degree, node degree of the basal ganglia (BG), and the primary motor cortex (PMC). Analyses were adjusted for potential confounders, notably age, sex, education level, MRI lag time, and wake time (model 1); additionally adjusted for diabetes status (model 2); and additionally adjusted for cardiovascular risk factors: BMI, office systolic blood pressure, antihypertensive medication, total-cholesterol-to-HDL-cholesterol ratio, lipid-modifying medication, alcohol use, smoking status, and history of CVD (model 3). *P* values < 0.05 were considered statistically significant. Interaction terms (e.g., HPA time × sex and HPA time × diabetes) were incorporated in the regression models to test for interaction among, on the one hand, physical activity and sedentary time and, on the other hand, sex and diabetes status, on node degree. For interaction terms a *P* value ≤ 0.10 was considered statistically significant.

## Results

### General characteristics of the study population

Table 1 shows the general characteristics of the study population stratified for low or high HPA. The study population consisted of 1715 participants, mean age was 59.6 ± 8.1 years, and 48% were women. The low HPA groups were older, had a higher BMI and waist circumference, more often had an adverse cardiovascular risk profile, and were more often smoker. Education levels did not differ significantly for individuals with different levels of physical activity (Table 1).

Table 2 shows the associations of physical activity and sedentary time with total brain volume and white matter volume. None of these associations remained significant after adjustment for demographics and cardiovascular risk factors. Participants without MRI or accelerometry data (*N* = 1736) had a higher BMI, more often had T2DM, a history of CVD, and mobility (Supplementary Table 1), compared with the study population.

**Table 2:** Associations of subtypes of PA and ST with volumes of the total brain and white matter

	Total brain volume		White matter volume	
	stβ (95% CI)	*P*	stβ (95% CI)	*P*
LPA time (high to low)				
Model 1	*− 0.019 (− 0.033, − 0.006)*	*0.005*	0.003 (− 0.019, 0.025)	0.784
Model 2	− 0.012 (− 0.025, 0.002)	0.092	0.011 (− 0.012, 0.033)	0.353
Model 3	− 0.008 (− 0.022, 0.005)	0.230	0.010 (− 0.013, 0.033)	0.395
HPA time (high to low)				
Model 1	− 0.013 (− 0.027, 0.001)	0.065	0.003 (− 0.020, 0.026)	0.821
Model 2	− 0.005 (− 0.019, 0.009)	0.506	0.011 (− 0.013, 0.034)	0.365
Model 3	− 0.003 (− 0.017, 0.011)	0.687	0.010 (− 0.014, 0.034)	0.423
Sedentary time (low to high)				
Model 1	*− 0.022 (− 0.037, − 0.008)*	*0.002*	− 0.008 (− 0.032, 0.016)	0.492
Model 2	− 0.013 (− 0.028, 0.001)	0.073	0.001 (− 0.024, 0.025)	0.963
Model 3	− 0.010 (− 0.024, 0.005)	0.200	0.001 (− 0.024, 0.026)	0.944

Associations between physical activity measures (minutes/day) with brain volumes. Regression coefficients and 95% CI indicate the mean difference in volume per SD. Higher LPA, HPA, or lower sedentary time. Model 1, adjusted for age, sex, education level, MRI lag time, wake time, and ICV. Model 2, additionally adjusted for diabetes status. Model 3, additionally adjusted for BMI, systolic blood pressure, antihypertensive medication, total-to-HDL-cholesterol ratio, lipid-modifying medication, smoking status, alcohol use, and history of cardiovascular disease

Italic values indicate *p*<0.05

**Fig. 2:**
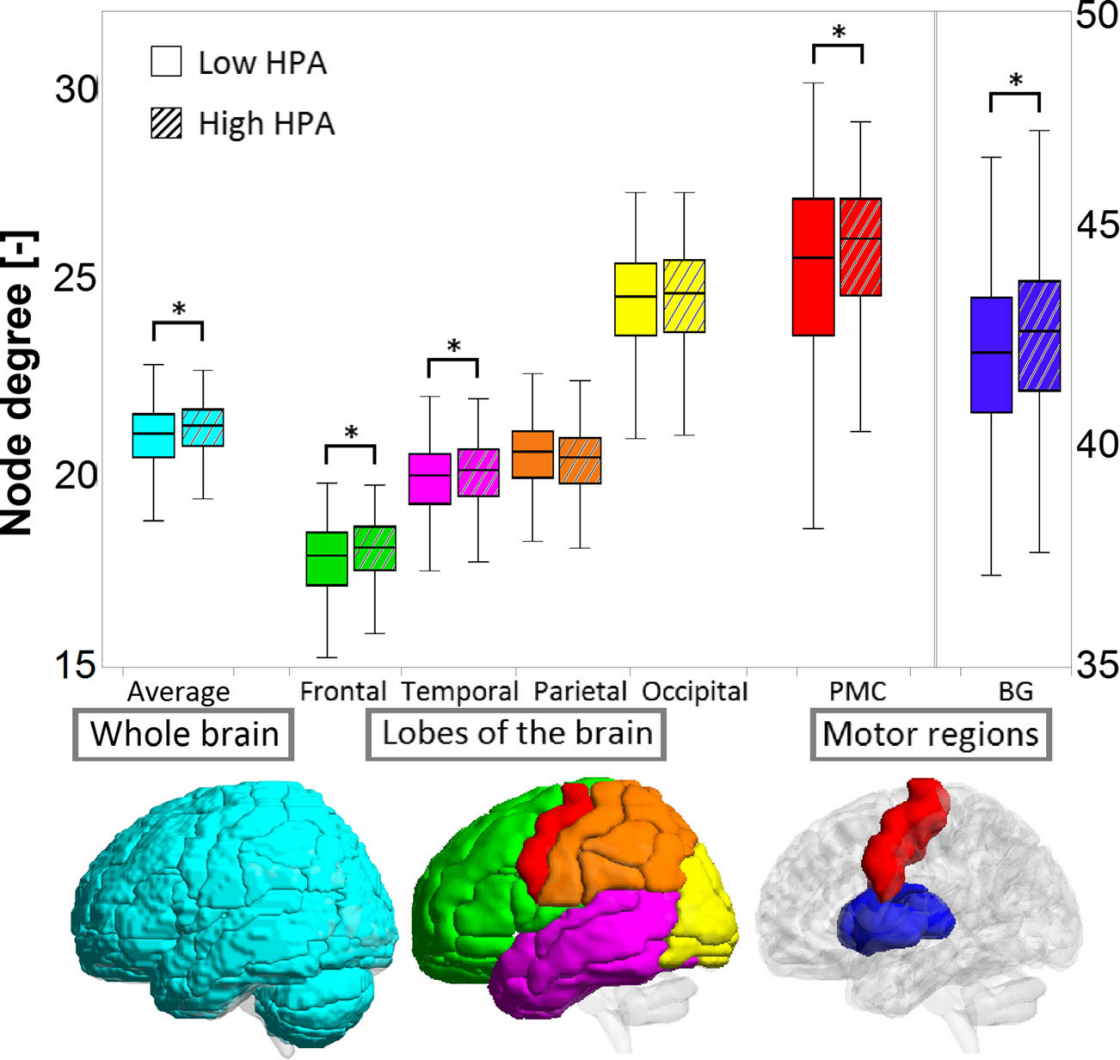
Boxplot for the node degree of the whole brain, the four lobes, and the motor regions of the brain for participants with high (dashed) and low HPA time. Note the different scale for the node degree of the basal ganglia (BG)

Figure 2 shows the node degree of the four lobes and specific motor regions for participants with high and low HPA. The low HPA group had a slightly, but significantly lower node degree of the frontal lobe (1.1%), temporal lobe (1.0%), basal ganglia (1.2%), and primary motor cortex (2.0%), compared with the high HPA group. The highest node degree was found in the basal ganglia because these structures are centrally located in the brain and therefore connected to many other regions (Fig. 1 A and B).

### Physical activity, sedentary behavior, and whole brain node degree

Table 3 shows the associations of physical activity and sedentary time with whole brain node degree. Lower levels of HPA and higher amount of sedentary time were associated with lower whole brain node degree in model 1. After adjustment for diabetes status, the association between sedentary time and whole brain node degree was attenuated (model 2). After full adjustment for cardiovascular risk factors, the association of HPA with whole brain node degree remained statistically significant (model 3).

**Table 3:** Associations of low- and high-intensity physical activity time and high sedentary time with whole brain node degree

	Whole brain node degree	
	stβ (95% CI)	*P* value
LPA time (high to low)		
Model 1	− 0.035 (− 0.081, 0.011)	0.137
Model 2	− 0.022 (− 0.069, 0.025)	0.356
Model 3	− 0.013 (− 0.061, 0.034)	0.580
HPA time (high to low)		
Model 1	*− 0.081 (− 0.128, − 0.033)*	*0.001*
Model 2	*− 0.068 (− 0.116, − 0.021)*	*0.005*
Model 3	*− 0.062 (− 0.112, − 0.013)*	*0.014*
Sedentary time (low to high)		
Model 1	*− 0.052 (− 0.101, − 0.002)*	*0.039*
Model 2	− 0.037 (− 0.087, 0.013)	0.152
Model 3	− 0.030 (− 0.081, 0.021)	0.250

Associations of physical activity measures with whole brain node degree. Standardized regression coefficients and 95% CI indicate the mean difference in node degree per SD lower physical activity and higher sedentary time. Model 1, adjusted for age, sex, education level, MRI lag time, and wake time. Model 2, additionally adjusted for diabetes status. Model 3, additionally adjusted for BMI, systolic blood pressure, antihypertensive medication, total-to-HDL-cholesterol ratio, lipid-modifying medication, smoking status, alcohol use, and history of cardiovascular disease

Italic values indicate *p*<0.05

Lower HPA was also associated with lower node degree of the temporal and frontal lobe in fully adjusted analyses but not with node degree of the parietal and occipital lobe (Table 4).

**Table 4:** Associations of low- and high-intensity physical activity time and high sedentary time with node degree of the frontal, temporal, parietal, and occipital lobe

	Degree		Degree		Degree		Degree	
	Frontal lobe		Temporal lobe		Parietal lobe		Occipital lobe	
	stβ (95% CI)	*P*	stβ (95% CI)	*P*	stβ (95% CI)	*P*	stβ (95% CI)	*P*
LPA time (high to low)								
Model 1	− 0.020 (− 0.067, 0.027)	0.420	− 0.031 (− 0.079, 0.017)	0.208	0.041 (− 0.007, 0.089)	0.097	0.016 (− 0.032, 0.065)	0.501
Model 2	− 0.001 (− 0.048, 0.047)	0.984	− 0.028 (− 0.077, 0.021)	0.266	0.034 (− 0.014, 0.083)	0.172	0.021 (− 0.028, 0.070)	0.397
Model 3	0.007 (− 0.042, 0.055)	0.786	− 0.027 (− 0.077, 0.024)	0.301	0.029 (− 0.021, 0.079)	0.262	0.019 (− 0.031, 0.069)	0.459
HPA time (high to low)								
Model 1	*− 0.076 (− 0.124, − 0.028)*	*0.002*	*− 0.068 (− 0.118, − 0.019)*	*0.007*	− 0.005 (− 0.055, 0.044)	0.835	− 0.001 (− 0.051, 0.048)	0.956
Model 2	*− 0.058 (− 0.106, − 0.009)*	*0.020*	*− 0.067 (− 0.117, − 0.016)*	*0.010*	− 0.014 (− 0.064, 0.036)	0.584	0.003 (− 0.047, 0.053)	0.909
Model 3	*− 0.054 (− 0.104, − 0.003)*	*0.038*	*− 0.066 (− 0.118, − 0.014)*	*0.014*	− 0.025 (− 0.077, 0.027)	0.347	− 0.002 (− 0.054, 0.051)	0.954
Sedentary time (low to high)								
Model 1	− 0.039 (− 0.089, 0.011)	0.126	− 0.032 (− 0.083, 0.020)	0.226	0.032 (− 0.019, 0.083)	0.227	0.004 (− 0.048, 0.055)	0.891
Model 2	− 0.017 (− 0.068, 0.034)	0.519	− 0.028 (− 0.082, 0.026)	0.296	0.024 (− 0.029, 0.071)	0.385	0.009 (− 0.044, 0.061)	0.745
Model 3	− 0.010 (− 0.062, 0.043)	0.721	− 0.028 (− 0.082, 0.026)	0.312	0.017 (− 0.036, 0.071)	0.535	0.003 (− 0.051, 0.058)	0.898

Associations of physical activity measures (minutes/day) with node degree. Regression coefficients and 95% CI indicate the mean difference in node degree per SD higher PA or lower sedentary time. Model 1, adjusted for age, sex, education level, MRI lag time, and wake time. Model 2, additionally adjusted for diabetes status. Model 3, additionally adjusted for BMI, systolic blood pressure, antihypertensive medication, total-to-HDL-cholesterol ratio, lipid-modifying medication, smoking status, alcohol use, and history of cardiovascular disease

Italic values indicate *p*<0.05

To put this into perspective, the difference in whole brain node degree associated with lower HPA time equivalent to 1 year of aging was 5.0 min less HPA time per day (Supplementary Table 6).

### Physical activity, sedentary behavior, and regional node degree

Table 5 shows the associations of physical activity and sedentary time with specific node degree of the motor regions, i.e., the basal ganglia and the primary motor cortex. Lower LPA and HPA and higher sedentary time were associated with lower node degree of the basal ganglia (model 1). These associations remained statistically significant after adjustment for diabetes status, except for higher sedentary time (model 2). Lower HPA remained statistically significantly associated with lower node degree of the basal ganglia after additional adjustment for cardiovascular risk factors (model 3). Lower HPA was also associated with a lower node degree of the primary motor cortex in model 1, but this association attenuated after adjustment for diabetes status and other cardiovascular risk factors (models 2 and 3). LPA and sedentary time were not associated with node degree of the primary motor cortex.

**Table 5:** Associations of low- and high-intensity physical activity time and high sedentary time with node degree of the basal ganglia and the primary motor cortex

	Degree		Degree	
	Basal ganglia		Primary motor cortex	
	stβ (95% CI)	*P*	stβ (95% CI)	*P*
LPA time (high to low)				
Model 1	*− 0.067 (− 0.116, − 0.019)*	*0.006*	− 0.038 (− 0.085, 0.010)	0.118
Model 2	*− 0.053 (− 0.102, − 0.004)*	*0.032*	− 0.017 (− 0.065, 0.031)	0.487
Model 3	− 0.049 (− 0.098, 0.001)	0.056	− 0.017 (− 0.066, 0.032)	0.489
HPA time (high to low)				
Model 1	*− 0.085 (− 0.134, − 0.035)*	*0.001*	− *0.052 (*− *0.101,* − *0.003)*	*0.037*
Model 2	*− 0.070 (− 0.121, − 0.020)*	*0.006*	− 0.030 (− 0.079, 0.019)	0.232
Model 3	*− 0.070 (− 0.121, − 0.018)*	*0.009*	− 0.035 (− 0.086, 0.016)	0.178
Sedentary time (low to high)				
Model 1	*− 0.052 (− 0.103, 0.000)*	*0.049*	− 0.033 (− 0.084, 0.017)	0.197
Model 2	− 0.034 (− 0.087, 0.018)	0.203	− 0.008 (− 0.060, 0.044)	0.761
Model 3	− 0.029 (− 0.082, 0.025)	0.295	− 0.008 (− 0.061, 0.045)	0.759

Associations of physical activity measures with node degree of the basal ganglia and primary motor cortex. Regression coefficients and 95% CI indicate the mean difference in node degree per SD lower physical activity and higher sedentary time. Model 1, adjusted for wake time, age, sex, education level, MRI lag time. Model 2, additionally adjusted for diabetes status. Model 3, additionally adjusted for BMI, systolic blood pressure, antihypertensive medication, total-to-HDL-cholesterol ratio, lipid-modifying medication, smoking status, alcohol use, and history of cardiovascular disease

Italic values indicate *p*<0.05

### Additional analyses

Qualitatively similar associations of physical activity and sedentary time with node degree were observed in a range of additional analyses: when we used total PA (Supplementary Table 2); when we used PA data measured with questionnaires instead of accelerometry (Supplementary Table 3); when model 3 was additionally adjusted for MRI quality, diet, (available in a smaller sample size), MMSE score, or white matter hyperintensity volume; and when we replaced BMI with waist circumference or replaced office with 24-h ambulatory blood pressure (available in a smaller sample size; Supplementary Tables 4 and 5). No interactions were observed with sex and diabetes status (*P*_interaction_ > 0.10 for all analyses).

## Discussion

In this study, we found an association of lower HPA with lower whole brain node degree, independent of major demographic, cardiovascular, and lifestyle risk factors. In other words, the difference in whole brain node degree associated with lower HPA time equivalent to one year of aging was 5.0 min less HPA time per day. LPA and ST were not significantly associated with whole brain node degree. In analyses on regional node degree, we found that lower HPA was associated with low node degree of the basal ganglia, but not of the primary motor cortex, independent of major demographic, cardiovascular, and lifestyle risk factors. LPA and sedentary time were not associated with markers of regional node degree.

We found that objectively measured lower HPA was associated with lower whole brain node degree. Our finding is in agreement with and extends a prior cross-sectional study, which observed an association between lower levels of self-reported physical activity and lower regional connectivity (nodal strength) in several frontal, parietal, and temporal brain regions [14] and lower local white matter organization (local efficiency). We also found an association of lower physical activity with lower node degree in the primary motor cortex (which is located in the frontal lobe), in other motor regions (i.e., the basal ganglia), and in the frontal and temporal lobes (Table 4). However, we did not find such an association for the parietal lobe. Our findings are also in line with a study, where lower aerobic fitness has been associated with lower structural connectivity in multiple cortical areas involving frontal, temporal, and motor regions [33].

We found no association of sedentary time with whole brain or regional node degree after adjustment for cardiovascular and lifestyle risk factors. This may mean that the association between sedentary time and regional connectivity is mediated by up- or downstream factors like BMI, blood pressure, lipid profile, prior cardiovascular disease, and/or lifestyle risk factors. In addition, our findings may indicate that potential prevention strategies for preservation of brain function on increasing physical activity, in particular HPA, may be more beneficial to the brain, as opposed to reduction of sedentary time. However, standardized regression coefficient was very similar for HPA and ST in model 1, which may indicate that the association between ST and connectivity is more strongly affected by mediating factors. Furthermore, reducing sedentary time might be easier to achieve than engaging in HPA. Therefore, studies on sedentary time remain of high relevance for the development of easy applicable prevention strategies. In addition, our study population consists of relatively healthy participants, with adequate cognitive function and relatively low cerebral small vessel disease load [29]. Therefore, analyses in more diseased populations may yield differential results.

In general, physical activity periodically increases blood flow to the brain, resulting in increased vascularization of the brain, improved supply of nutrients, and removal of metabolic waste. More specifically, higher levels of physical activity or cardiorespiratory fitness have been associated with an increase in cerebral perfusion and cerebral oxygen supply to the prefrontal cortex [6], which is an area involved in motor function [10]. Higher levels of physical activity stimulate processes of neurogenesis (formation of new neurons) and angiogenesis (formation of blood vessels) and reduce inflammation [24]. Furthermore, being physically active has been shown to reduce cardiovascular risk factors such as type 2 diabetes and hypertension through increasing neurotrophic factors, reducing oxidative stress, and/or reducing beta-amyloid formation [39]. These mechanisms may reduce the incidence of mild cognitive impairment and dementia [9, 13].

Strengths of this study are the large sample size, the population-based design, the objective measurement of physical activity and sedentary behavior, the extensive assessment of potential confounders, which enabled us to substantially reduce potential residual confounding, and the broad array of additional analyses, which all gave consistent results. Furthermore, the use of diffusion MRI scans to study structural connectivity measures enabled us to find more subtle brain alterations, as we did not find associations with brain volumes in this population. The large number of diffusion MRI scans was semi-automatically processed blinded to group status, which ensures an objective analysis.

There are also some limitations. First, the cross-sectional design of the study implies that any conclusion about causality should be made with caution. Further longitudinal studies and intervention trials may be prompted to investigate whether specific connectivity loss can be reversed or prevented by increasing high-intensity physical activity. Second, the study population was oversampled for type 2 diabetes. However, analyses were adjusted for diabetes status, and no consistent interaction with diabetes status was observed. Third, participants with missing data had a more adverse cardiovascular risk profile, which might have led to an underestimation of our findings. Fourth, although we cannot exclude the possibility of residual confounding by variables not included in this study, we have adjusted for many known confounding factors, which may have resulted in overadjustment and thus underestimate the association of physical activity and sedentary time with node degree.

We showed that objectively measured lower HPA, but not LPA and ST, was associated with lower whole brain node degree and node degree in specific brain regions highly specialized in motor function. Further research is needed to establish whether more HPA may preserve structural brain connectivity.

## Electronic supplementary material

ESM 1 (DOCX 617 kb)

## Data Availability

The data of this study derive from The Maastricht Study, but restrictions apply to the availability of these data, which were used under license for the current study. Data are however available from the authors upon reasonable request and with permission of The Maastricht Study management team.
