# Supplementary material for: Association of physical activity and sedentary time with structural brain networks—The Maastricht Study
Source: GeroScience. 2020 Oct 9;43(1):239–52. doi: 10.1007/s11357-020-00276-z (PMC8050169; doi:10.1007/s11357-020-00276-z)
Supplement: Supplementary file 1 — (DOCX 617 kb) [file 11357_2020_276_MOESM1_ESM.docx]

# Supplementary material

1. Detailed protocols of the measurements performed within The Maastricht Study:

*Questionnaires.* As described elsewhere^1^, we used web-based questionnaires to obtain information regarding smoking status (never/former/current), alcohol consumption, educational level, physical activity, diet, prior CVD and cognitive impairment. Alcohol consumption was classified as none, low (1– 7 glasses/wk for women and 1–14 glasses/wk for men) and high (> 7 glasses/wk for women and >14 glasses/wk for men). Educational level was classified as low (no education, primary education, lower vocational education), intermediate (intermediate general secondary education, intermediate vocational education, higher general secondary education), or high (higher vocational education or university). Diet was assessed by a tailor-made FFQ developed by use of the National FFQ Tool. Prior CVD was defined as a history of myocardial infarction; stroke; or vascular surgery (including angioplasty) on coronary, carotid, abdominal aortic, or peripheral arteries based on the Rose questionnaire. Cognitive impairment was measured using the Mini-Mental State Examination (MMSE). Medication use was assessed in a medication interview where generic name, dose, and frequency were registered. Subjective physical activity was assessed by means of a modified version of the Champs questionnaire.

*Laboratory assessments.* Plasma glucose is measured with a standard enzymatic hexokinase reference method, and serum total cholesterol, HDL cholesterol, and triglycerides are measured with standard (enzymatic and/or colorimetric) methods by an automatic analyzer (until 9 May 2012: Beckman Synchron LX20, Beckman Coulter Inc., Brea, USA; after 9 May 2012: Cobas 6000, Roche diagnostics, Mannheim, Germany). When appropriate LDL cholesterol is calculated according to the Friedewald formula^2^. HbA1c is measured with ion-exchange high performance liquid chromatography (HPLC) (Variant tm II, Bio-Rad, Hercules, California, USA).

*Glucose metabolism status.* To determine glucose metabolism status, all participants, except those who used insulin, underwent a standardized 2-h 75g oral glucose tolerance test (OGTT) after an overnight fast. For safety reasons, participants with a fasting glucose level above 11·0 mmol/L, as determined by a finger prick, did not undergo the OGTT. For these individuals, fasting glucose level and information about diabetes medication were used to determine glucose metabolism status. Glucose metabolism status was defined according to the WHO 2006 criteria into NGM, prediabetes, and T2DM^3^. Participants were considered to have T2DM if they had a fasting blood glucose (FBG) $\geq$7.0 mmol/l, or a 2hr post-load blood glucose $\geq$11.1 mmol/l or used oral glucose-lowering medication or insulin, prediabetes if they had a FBG $\geq$6.1 mmol/l and/or a 2hr post-load blood glucose $\geq$7.8 mmol/l, and NGM if they had a FBG <6.1 mmol/l, and a 2hr post-load blood glucose <7.8 mmol/l and no use of diabetes medication. Participants with type 1 diabetes or other types of diabetes were excluded from the analysis.

*Physical examination.* Weight and height are measured without shoes and wearing light clothing using a scale and stadiometer to the nearest 0.5 kg or 0.1 cm (Seca, Hamburg, Germany). Waist circumference is measured with a flexible plastic tape measure (Seca, Hamburg, Germany) in a duplicate midway between the lower rib margin and the iliac crest at the end of expiration, to the nearest 0.5 cm.

*Blood pressure.* Office blood pressure is determined three times on the right arm after a 10-minute rest period, using a non-invasive blood pressure monitor (Omron 705IT, Japan). When the difference between measurement two and three is more than 10 mmHg, a fourth measurement is performed. All available measurements are used to calculate the average blood pressure. Ambulatory 24-h blood pressure (WatchBP O3, Microlife, Switzerland, respectively) is measured at the non-dominant arm, using an ambulatory device that is programmed to take blood pressure readings every 15 minutes from 8.00 – 23.00 and every 30 minutes from 23.00 – 8.00.

1. Detailed methods for calculation of reference networks and sparsity:

In structural connectivity analysis, connectivity matrices are based on tractography. As a result, not all pairs of nodes will have white matter connections, which leads to ‘sparse’ connectivity matrices. The measure sparsity reflects the extent of a graph’s deviation from the corresponding fully connected graph. However, this differs between subjects and is also influenced by scan quality. Therefore, thresholding is applied, which ensures that each subject has the same number of nodes and edges. The reference network was calculated from all individual binarized connectivity matrices, for a specific subset of participants (e.g., the physical activity network was based on the participants who met the physical activity guidelines). This is illustrated in a simplified example, not a real network (Supplementary Figure 3). First, all individual binarized connectivity matrices were summed (Step 1 in Supplementary Figure 3). This reference network was proportionally thresholded to a sparsity of 0.80 (Step 2 in Supplementary Figure 3). This means that the 20% connections that were present in most of the participants were selected, in other words, the 20% of the nodes with the highest sum score. These 20% nodes were used to create a binary mask (Step 3 in Supplementary Figure 3) that was used to select for each participant a weighted, undirected network with a sparsity, close to the sparsity of the reference network (Step 4 in Supplementary Figure 3). The actual sparsity in the individuals is a bit higher than 0.80, because not each individual has a white matter tract at each connection in the reference network.


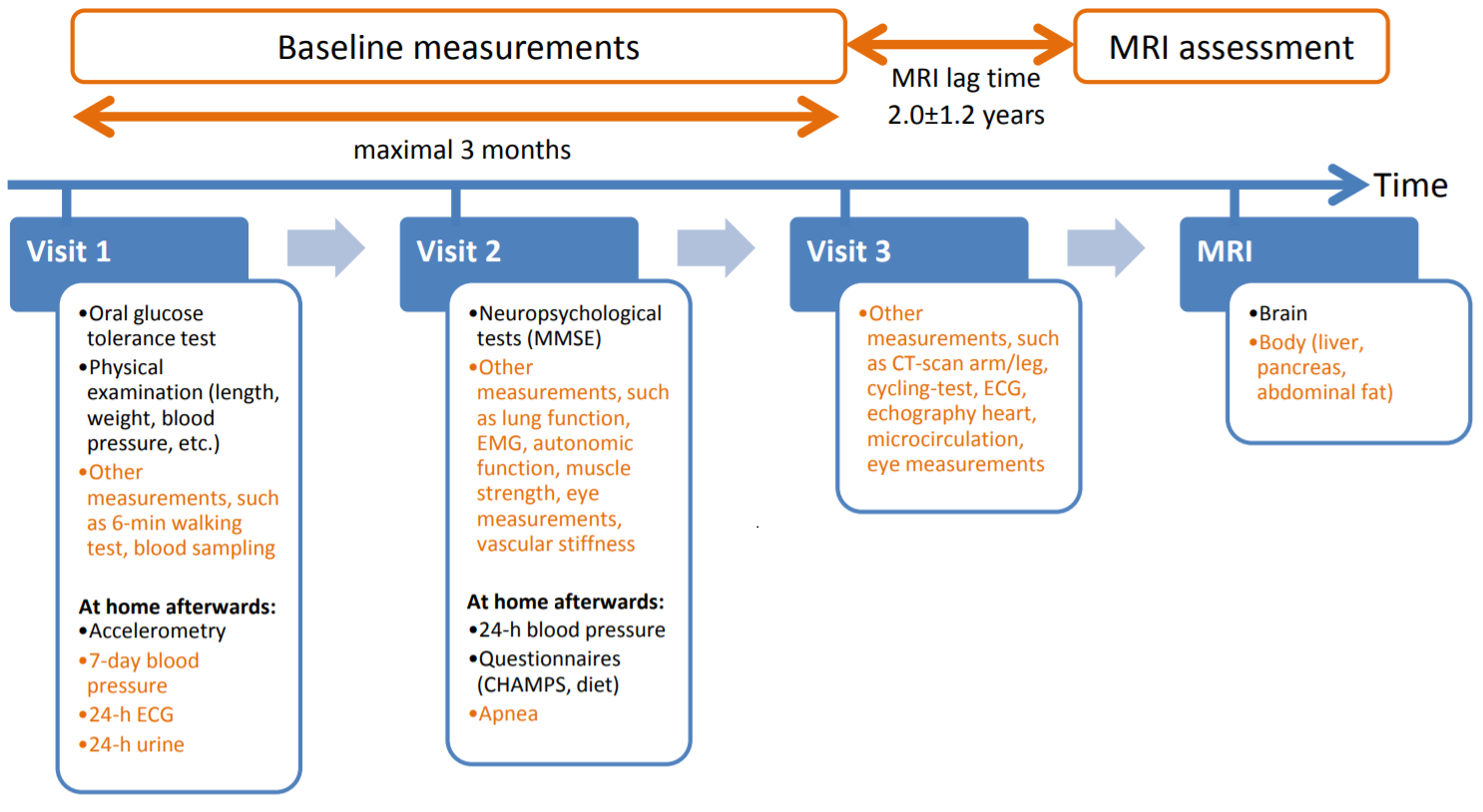


Supplementary Figure 1: Detailed time line of the measurements in The Maastricht Study. Measurements in orange were not used in this study.


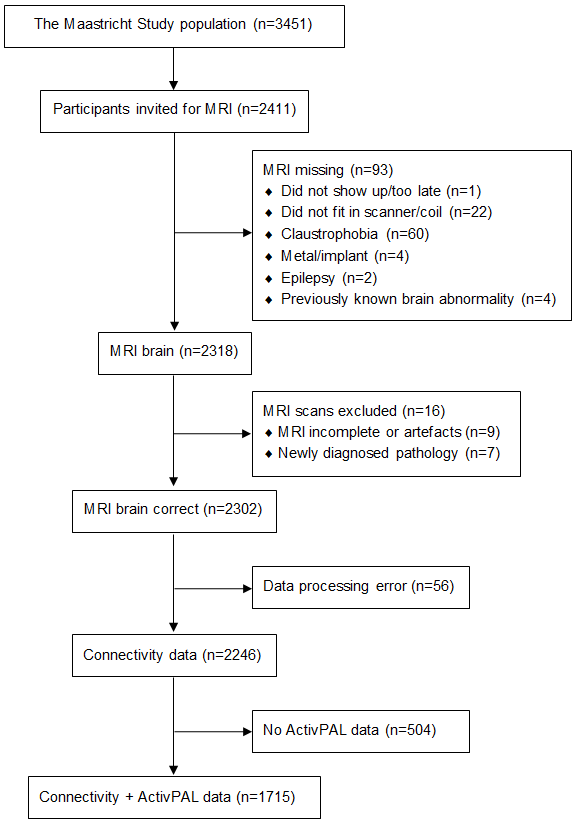


Supplementary Figure 2: Flowchart of the study population.

*
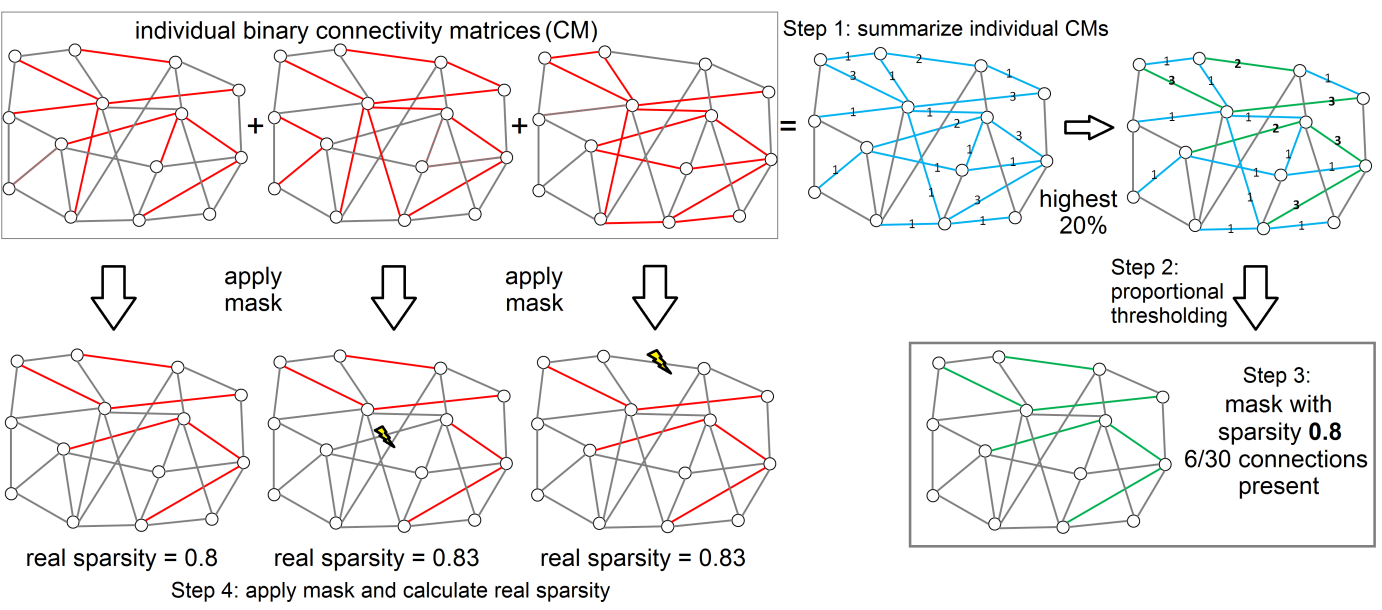
*

Supplementary Figure 3: Proportional thresholding. The red edges indicate the edges present in the individual networks. The blue edges in Step 1 indicate the edges present in a least one of the individual networks and the corresponding number indicates in how many individual networks this edges was present. The green edges in Step 2 indicate the 20% of edges with the highest occurrence over the individual networks, resulting in the reference network depicted in Step 3.

**Supplementary Table 1:** General characteristics of participants stratified by availability of physical activity and/or brain MRI data.

| **Characteristic** | **Total**  **(n=3451)** | **Physical activity and MRI data available**  **(n=1715)** | **Physical activity and/or MRI data not available**  **(n=1736)** | ***P*** |
| --- | --- | --- | --- | --- |
| **Demographics** |  |  |  |  |
| Age (years) | 59.8±8.3 | 59.6±8.1 | 60.0±8.5 | 0.183 |
| Sex, female (%) | 48.6 | 48.4 | 48.7 | 0.843 |
| Education level (%), Low/Middle/High | 33.6/28.2/38.2 | 30.5/29.0/40.5 | 36.6/27.5/35.9 | <0.001 |
| **Cardiovascular risk factors** |  |  |  |  |
| BMI (kg/m^2^) | 27.1±4.6 | 26.6±4.2 | 27.5±4.8 | <0.001 |
| Waist circumference (cm) | 95.9±13.8 | 94.4±12.7 | 97.5±14.6 | <0.001 |
| Systolic blood pressure (mmHg) | 135.1±18.2 | 134.0±17.3 | 136.1±19.0 | 0.001 |
| Diastolic blood pressure (mmHg) | 76.1±9.9 | 76.1±9.7 | 76.2±10 | 0.634 |
| T2DM (%) | 23.0 | 23.7 | 20.9 | <0.001 |
| Hypertension, yes (%) | 56.4 | 53.3 | 59.4 | <0.001 |
| Total cholesterol-to-HDL-ratio | 3.6±1.2 | 3.6±1.1 | 3.9±1.3 | <0.001 |
| History of CVD, yes (%) | 16.7 | 12.7 | 20.7 | <0.001 |
| **Medication use** |  |  |  |  |
| Diabetes medication, yes (%) | 19.7 | 18.0 | 60.6 | <0.001 |
| Antihypertensive medication, yes (%) | 35.1 | 36.1 | 32.1 | 0.079 |
| Lipid-modifying medication, yes (%) | 31.1 | 31.5 | 30.1 | 0.546 |
| **Lifestyle factors** |  |  |  |  |
| Alcohol (%), None/Low/High | 18.6/55.4/26.0 | 17.0/57.2/25.9 | 20.2/53.7/26.1 | 0.001 |
| Smoking (%), Never/Former/Current | 34.5/51.6/13.8 | 38.0/49.8/12.3 | 31.1/51.9/15.4 | <0.001 |
| **Cognitive score** |  |  |  |  |
| MMSE total score | 28.9±1.3 | 29.0±1.2 | 28.9±1.4 | <0.001 |
| **Other** |  |  |  |  |
| MRI lag time | 2.3±1.3 | 2.0±1.2 | 2.9±1.4* | <0.001 |
| Data are presented as means ± standard deviation, or percentages for categorical variables.  T2DM, indicates type 2 diabetes mellitus; HDL, high-density lipoprotein; CVD, cardiovascular disease;  MMSE, Mini-Mental State Examination. *n=699 (physical activity data not available). | | | |  |

Supplementary Table 2a: Associations of total physical activity time with whole brain node degree and node degree of the basal ganglia and primary motor cortex.

|  | Whole brain  node degree |  | Degree  Basal Ganglia |  | Degree  Primary Motor Cortex |  |
| --- | --- | --- | --- | --- | --- | --- |
| *TPA* *time*  *(high to low)* | stβ (95% CI) | *P* | stβ (95% CI) | *P* | stβ (95% CI) | *P* |
| Model 1 | **-0.064 (-0.111, -0.018)** | **0.007** | **-0.093 (-0.142, -0.044)** | **<0.001** | **-0.054 (-0.102, -0.006)** | **0.027** |
| Model 2 | **-0.049 (-0.097, -0.002)** | **0.042** | **-0.077 (-0.127, -0.027)** | **0.003** | -0.028 (-0.077, 0.021) | 0.265 |
| Model 3 | -0.041 (-0.090, 0.009) | 0.111 | **-0.075 (-0.127, -0.023)** | **0.005** | -0.031 (-0.083, 0.020) | 0.231 |
| Associations of total physical activity (minutes/day) with node degree. Regression coefficients and 95% CI indicate the mean difference in node degree per SD higher TPA. Model 1: Adjusted for age, sex, education level, MRI lag time, and wake time. Model 2: additionally adjusted for diabetes status. Model 3: additionally adjusted for BMI, systolic blood pressure, antihypertensive medication, total-to-HDL-cholesterol-ratio, lipid-modifying medication, smoking status, alcohol use, and history of cardiovascular disease. | | | | | | |

Supplementary Table 2b: Associations of total physical activity time with total brain and white matter volume.

|  | Total brain volume |  | WM volume |  |
| --- | --- | --- | --- | --- |
| *TPA time (high to low)* | stβ (95% CI) | *P* | stβ (95% CI) | *P* |
| Model 1 | **-0.022 (-0.035, -0.008)** | **0.001** | 0.004 (-0.019, 0.026) | 0.746 |
| Model 2 | -0.012 (-0.026, 0.012) | 0.092 | 0.014 (-0.009, 0.037) | 0.241 |
| Model 3 | -0.009 (-0.024, 0.005) | 0.189 | 0.013 (-0.011, 0.037) | 0.280 |
| Associations between physical activity measures (minutes/day) with brain volumes.  Regression coefficients and 95% CI indicate the mean difference in volume per SD  higher TPA time. Model 1: Adjusted for age, sex, education level, MRI lag time, wake time,  and ICV. Model 2: additionally adjusted for diabetes status. Model 3: additionally  adjusted for BMI, systolic blood pressure, antihypertensive medication,  total-to-HDL-cholesterol-ratio, lipid-modifying medication, smoking status, alcohol use,  and history of cardiovascular disease. | | | | |

Supplementary Table 2c: Associations of total physical activity time with node degree of the frontal, temporal, parietal and occipital lobe.

|  | Degree  Frontal lobe | Degree  Temporal lobe | Degree  Parietal lobe | Degree  Occipital lobe |
| --- | --- | --- | --- | --- |
| TPA time (high to low) | stβ (95% CI) | stβ (95% CI) | stβ (95% CI) | stβ (95% CI) |
| Model 1 | **-0.050 (-0.098, -0.003)** | **-0.056 (-0.105, -0.007)** | 0.031 (-0.017, 0.080) | 0.013 (-0.036, 0.062) |
| Model 2 | -0.027 (-0.075, 0.022) | **-0.054 (-0.104, -0.003)** | 0.022 (-0.027, 0.072) | 0.019 (-0.031, 0.069) |
| Model 3 | -0.019 (-0.070, 0.032) | **-0.054 (-0.106, -0.001)** | 0.014 (-0.038, 0.066) | 0.016 (-0.037, 0.068) |
| Associations of physical activity measures (minutes/day) with node degree. Regression coefficients and 95% CI indicate the mean difference in node degree per SD higher PA time. Model 1: Adjusted for age, sex, education level, MRI lag time, and wake time. Model 2: additionally adjusted for diabetes status. Model 3: additionally adjusted for BMI, systolic blood pressure, antihypertensive medication, total-to-HDL-cholesterol-ratio, lipid-modifying medication, smoking status, alcohol use, and history of cardiovascular disease. | | | | |

Supplementary Table 3: Associations of physical activity time determined with CHAMPS questionnaire with whole brain node degree and node degree of the basal ganglia and primary motor cortex.

|  | Whole brain  node degree |  | Degree  Basal Ganglia |  | Degree  Primary Motor Cortex |  |
| --- | --- | --- | --- | --- | --- | --- |
| *Total PA* *time*  *(high to low)* | stβ (95% CI) | *P* | stβ (95% CI) | *P* | stβ (95% CI) | *P* |
| Model 1 | -0.032 (-0.081, 0.016) | 0.188 | **-0.055 (-0.106, -0.004)** | **0.033** | **-0.069 (-0.119, -0.020)** | **0.006** |
| Model 2 | -0.023 (-0.072, 0.025) | 0.343 | -0.045 (-0.096, 0.006) | 0.081 | **-0.056 (-0.106, -0.006)** | **0.027** |
| Model 3 | -0.019 (-0.068, 0.029) | 0.433 | -0.041 (-0.092, 0.010) | 0.113 | **-0.057 (-0.107, -0.007)** | **0.025** |
| *MVPA* *time*  *(high to low)* | stβ (95% CI) | *P* | stβ (95% CI) | *P* | stβ (95% CI) | *P* |
| Model 1 | **-0.065 (-0.111, -0.018)** | **0.007** | **-0.104 (-0.153, -0.055)** | **<0.001** | **-0.102 (-0.150, -0.054)** | **<0.001** |
| Model 2 | **-0.054 (-0.101, -0.007)** | **0.026** | **-0.093 (-0.142, -0.044)** | **<0.001** | **-0.087 (-0.135, -0.038)** | **<0.001** |
| Model 3 | **-0.049 (-0.097, 0.001)** | **0.043** | **-0.075 (-0.140, -0.040)** | **<0.001** | **-0.091 (-0.140, -0.041)** | **<0.001** |
| Associations of physical activity (CHAMPS questionnaire) with node degree. Regression coefficients and 95% CI indicate the mean difference in node degree per SD higher PA. Model 1: Adjusted for age, sex, education level, and MRI lag time. Model 2: additionally adjusted for diabetes status. Model 3: additionally adjusted for BMI, systolic blood pressure, antihypertensive medication, total-to-HDL-cholesterol-ratio, lipid-modifying medication, smoking status, alcohol use, and history of cardiovascular disease. MVPA indicates mild-to-vigorous physical activity (hours/week). CHAMPS questionnaire information available in n=1486. | | | | | | |

Supplementary Table 4: Associations of low and high-intensity physical activity time, and high sedentary time with whole brain node degree, additionally corrected for waist circumference, 24h blood pressure, MRI quality, diet, or white matter hyperintensity volume.

|  | Whole brain node degree |  |
| --- | --- | --- |
| *LPA time (high to low)* | stβ (95% CI) | *P-value* |
| Model 3 | -0.013 (-0.061, 0.034) | 0.580 |
| Model 3 + waist circ. for BMI | -0.012 (-0.060, 0.036) | 0.621 |
| Model 3 + 24h for office BP | -0.010 (-0.062, 0.042) | 0.705 |
| Model 3 + MRI quality | -0.007 (-0.052, 0.038) | 0.757 |
| Model 3 + DHD score | -0.008 (-0.057, 0.041) | 0.736 |
| Model 3 + WMH* + ICV | -0.008 (-0.056, 0.039) | 0.725 |
| *HPA time (high to low)* | stβ (95% CI) | *P-value* |
| Model 3 | **-0.062 (-0.112, -0.013)** | **0.014** |
| Model 3 + waist circ. for BMI | **-0.056 (-0.107, -0.006)** | **0.027** |
| Model 3 + 24h for office BP | **-0.070 (-0.124, -0.015)** | **0.012** |
| Model 3 + MRI quality | **-0.054 (-0.102, -0.007)** | **0.023** |
| Model 3 + DHD score | **-0.064 (-0.115, -0.014)** | **0.013** |
| Model 3 + WMH* + ICV | **-0.061 (-0.110, -0.012)** | **0.015** |
|  |  |  |
| *Sedentary time (low to high)* | stβ (95% CI) | *P-value* |
| Model 3 | -0.030 (-0.081, 0.021) | 0.250 |
| Model 3 + waist circ. for BMI | -0.019 (-0.070, 0.033) | 0.478 |
| Model 3 + 24h for office BP | -0.026 (-0.083, 0.030) | 0.362 |
| Model 3 + MRI quality | -0.019 (-0.068, 0.030) | 0.448 |
| Model 3 + DHD score | -0.022 (-0.075, 0.031) | 0.414 |
| Model 3 + WMH* + ICV | -0.025 (-0.076, 0.026) | 0.327 |
| Associations of physical activity measures (minutes/day) with whole brain node degree.  Standardized regression coefficients and 95% CI indicate the mean difference in node  degree per SD lower physical activity and higher sedentary time.  Model 3: Adjusted for age, sex, education level, MRI lag time, wake time, diabetes status, BMI,  systolic blood pressure, antihypertensive medication, total-to-HDL-cholesterol-ratio,  lipid-modifying medication, smoking status, alcohol use, and history of cardiovascular  disease. Waist circ. Indicated waist circumference; 24h BP, 24-hour ambulatory blood  pressure; DHD, Dutch Healthy Diet index; WMH, white matter hyperintensity volume;  ICV, intracranial volume. 24h BP available in n=1408. MRI quality available  in n=1657. DHD score available in n=1581. *log-transformed WMH volume. | | |

Supplementary Table 5: Associations of low- and high-intensity physical activity time, and high sedentary time with node degree of the basal ganglia and the primary motor cortex.

|  | Degree  Basal Ganglia |  | Degree  Primary Motor Cortex |  |
| --- | --- | --- | --- | --- |
| *LPA time (high to low)* | stβ (95% CI) |  | stβ (95% CI) |  |
| Model 3 | -0.049 (-0.098, 0.001) | 0.056 | -0.017 (-0.066, 0.032) | 0.489 |
| Model 3 + waist circ. for BMI | -0.048 (-0.098, 0.002) | 0.059 | -0.017 (-0.066, 0.032) | 0.490 |
| Model 3 + 24h for office BP | **-0.062 (-0.116, -0.009)** | **0.022** | -0.015 (-0.068, 0.038) | 0.574 |
| Model 3 + MRI quality | -0.045 (-0.094, 0.004) | 0.074 | -0.018 (-0.067, 0.031) | 0.475 |
| Model 3 + DHD score | -0.044 (-0.096, 0.007) | 0.092 | -0.005 (-0.056, 0.046) | 0.838 |
| *HPA time (high to low)* | stβ (95% CI) |  | stβ (95% CI) |  |
| Model 3 | **-0.070 (-0.121, -0.018)** | **0.009** | -0.035 (-0.086, 0.016) | 0.178 |
| Model 3 + waist circ. for BMI | **-0.066 (-0.119, -0.014)** | **0.014** | -0.035 (-0.087, 0.016) | 0.181 |
| Model 3 + 24h for office BP | **-0.080 (-0.136, -0.023)** | **0.006** | -0.052 (-0.108, 0.004) | 0.068 |
| Model 3 + MRI quality | **-0.066 (-0.117, -0.014)** | **0.012** | -0.036 (-0.087, 0.015) | 0.172 |
| Model 3 + DHD score | **-0.069 (-0.112, -0.016)** | **0.011** | -0.033 (-0.086, 0.020) | 0.219 |
|  |  |  |  |  |
| *Sedentary time (low to high)* | stβ (95% CI) |  | stβ (95% CI) |  |
| Model 3 | -0.029 (-0.082, 0.025) | 0.295 | -0.008 (-0.061, 0.045) | 0.759 |
| Model 3 + waist circ. for BMI | -0.023 (-0.077, 0.031) | 0.402 | -0.008 (-0.061, 0.045) | 0.766 |
| Model 3 + 24h for office BP | -0.036 (-0.094, 0.023) | 0.233 | -0.019 (-0.076, 0.039) | 0.531 |
| Model 3 + MRI quality | -0.025 (-0.078, 0.028) | 0.358 | -0.009 (-0.062, 0.044) | 0.738 |
| Model 3 + DHD score | -0.020 (-0.075, 0.036) | 0.482 | 0.002 (-0.052, 0.057) | 0.930 |
| Associations of physical activity measures with node degree of the basal ganglia and primary motor cortex. Regression coefficients and95% CI indicate the mean difference in node degree per SD lower physical activity and higher sedentary time. Model 3: wake time, age, sex, education level, MRI lag time, diabetes status, BMI, systolic blood pressure, antihypertensive medication, total-to-HDL-cholesterol-ratio, lipid-modifying medication, smoking status, alcohol use, and history of cardiovascular disease. 24h BP available in n=1408. MRI quality available  in n=1657. DHD score available in n=1581. | | | | |

**Supplementary Table 6**: Relative change for HPA time and age in whole brain node degree.

|  | **Node degree^*^** |  |
| --- | --- | --- |
|  | **standardized β** | **p-value** |
| *HPA time* | 0.081 | 0.001 |
| *Age* | -0.177 | <0.001 |
| $\frac{st\beta_{age}}{st\beta_{HPA}}\cdot\frac{SD_{HPA}}{SD_{age}}$ | **5.0 years** |  |
| Adjusted for age, sex, education level, MRI lag time, and wake time. | | |

# References

1. Schram MT, Sep SJ, van der Kallen CJ, et al. The Maastricht Study: an extensive phenotyping study on determinants of type 2 diabetes, its complications and its comorbidities. *Eur J Epidemiol.* 2014;29(6):439-451.

2. Friedewald WT, Levy RI, Fredrickson DS. Estimation of the concentration of low-density lipoprotein cholesterol in plasma, without use of the preparative ultracentrifuge. *Clin Chem.* 1972;18(6):499-502.

3. Organization WH. *Definition and diagnosis of diabetes mellitus and intermediate hyperglycemia: report of a WHO/IDF consultation.* Geneva, Switzerland2006.
